# Augmented reality visualization in brain lesions: a prospective randomized controlled evaluation of its potential and current limitations in navigated microneurosurgery

**DOI:** 10.1007/s00701-021-05045-1

**Published:** 2021-12-13

**Authors:** Anna L. Roethe, Judith Rösler, Martin Misch, Peter Vajkoczy, Thomas Picht

**Affiliations:** 1 Department of Neurosurgery, Charité-Universitätsmedizin Berlin, Berlin, Germany; 2 Cluster of Excellence Matters of Activity, Image Space Material, Humboldt-Universität Zu Berlin, Berlin, Germany; 3 Berlin Simulation and Training Center (BeST), Charité-Universitätsmedizin Berlin, Berlin, Germany

**Keywords:** Augmented reality, Brain tumors, Intraoperative visualization, Navigated microscope

## Abstract

**Background:**

Augmented reality (AR) has the potential to support complex neurosurgical interventions by including visual information seamlessly. This study examines intraoperative visualization parameters and clinical impact of AR in brain tumor surgery.

**Methods:**

Fifty-five intracranial lesions, operated either with AR-navigated microscope (*n* = 39) or conventional neuronavigation (*n* = 16) after randomization, have been included prospectively. Surgical resection time, duration/type/mode of AR, displayed objects (*n*, type), pointer-based navigation checks (*n*), usability of control, quality indicators, and overall surgical usefulness of AR have been assessed.

**Results:**

AR display has been used in 44.4% of resection time. Predominant AR type was navigation view (75.7%), followed by target volumes (20.1%). Predominant AR mode was picture-in-picture (PiP) (72.5%), followed by 23.3% overlay display. In 43.6% of cases, vision of important anatomical structures has been partially or entirely blocked by AR information. A total of 7.7% of cases used MRI navigation only, 30.8% used one, 23.1% used two, and 38.5% used three or more object segmentations in AR navigation. A total of 66.7% of surgeons found AR visualization helpful in the individual surgical case. AR depth information and accuracy have been rated acceptable (median 3.0 vs. median 5.0 in conventional neuronavigation). The mean utilization of the navigation pointer was 2.6 × /resection hour (AR) vs. 9.7 × /resection hour (neuronavigation); navigation effort was significantly reduced in AR (*P* < 0.001).

**Conclusions:**

The main benefit of HUD-based AR visualization in brain tumor surgery is the integrated continuous display allowing for pointer-less navigation. Navigation view (PiP) provides the highest usability while blocking the operative field less frequently. Visualization quality will benefit from improvements in registration accuracy and depth impression.

**German clinical trials registration number.:**

DRKS00016955.

**Supplementary Information:**

The online version contains supplementary material available at 10.1007/s00701-021-05045-1.

## Introduction

The increase of visual information provided during neurosurgical procedures poses the threat of unwanted interference and cognitive overload for the surgeon. While the history of augmented reality (AR) visualization in neurosurgery already began in 1986 [16, 39, 42], subsequent years of technological innovation have been dominated by more prominent clinical developments in frameless neuronavigation, which is now a widely established technique to guide the intervention [17, 23, 32, 43, 45]. Conventional neuronavigation introduced the separate navigation display as a “second screen” into the operating room (OR), necessitating the exchange of surgical instruments and a dedicated navigation pointer on the one hand, as well as alternate viewing directions between the surgical site and the extra display on the other hand. The demand for the inclusion of surgically relevant information directly into the surgical field of view has been discussed ever since [1, 20, 42]. Consequently, several types of AR technology have been subject to both preclinical and early clinical investigation, such as image projection techniques, additional head-up or head-mounted displays (HUD, HMD), tablet- or monitor-based systems, and image injection into the surgical microscope [8–11, 15, 16, 18, 24, 26, 27, 29, 35]. AR includes a real-world view (i.e., the surgical site) as the main visual reference plane, which is augmented by an overlay of digital virtual information typically provided by volumetric imaging (CT, MRI, functional information) [36, 42]. Thereby, AR integration of surgically relevant information can provide a situated visualization [21], i.e., a virtual manifestation of the surgeon’s mental projections — such as tumor borders, adjacent risk structures — applied to the surgical area. Integrating the overlay at the correct position, scale and orientation mark the ideal of AR visualization. Potential benefits include reduced surgical risk and the reduction of intraoperative cognitive load as well as the increased availability of detailed visual representations for the whole surgical team. While many applications continue to be limited to research only [11, 20, 24], commercial software development focused on the integration of AR functionalities into the surgical microscope [14, 40], thus making the navigated microscope the most popular and most easily available sub-modality of AR in neurosurgery today [25, 31, 33–35]. Its clinical feasibility and overall usefulness in the areas of skin incision planning, craniotomy, subsurface lesion targeting, and risk management across the neurosurgical subspecialties have been claimed in recent studies [2–7, 22, 28, 33, 36, 41, 44]. However, while intraoperative utilization of AR is increasingly discussed as beneficial, the exact impact of AR-guided interventions on surgical decision-making, intraoperative workflow, and patient outcome still remains unclear. Besides challenges in registration accuracy, which is a known limitation up to the current software generation [13, 38], the aspects of visualization quality in particular proved to be one limiting factor to broader clinical application [16, 25, 42]. This study addresses the integration of current generation AR into the clinical routine by the example of the navigated operating microscope, aiming to provide a detailed overview of the predominant requirements in intraoperative data visualization. The study further analyzes the application in brain tumor surgery and compares HUD-based AR guidance with conventionally navigated interventions.

## Methods and materials

In order to assess both the qualitative dimensions of the AR visualization at a given point during surgery and the quantifiable parameters indicating its overall usefulness, we applied a mixed methods study design involving direct participant observation in the operating room (OR) using a checklist specifically designed for this purpose, analysis of clinical data and microscope recordings, complemented by user interviews.

### Technical setup

**Fig. 1:**
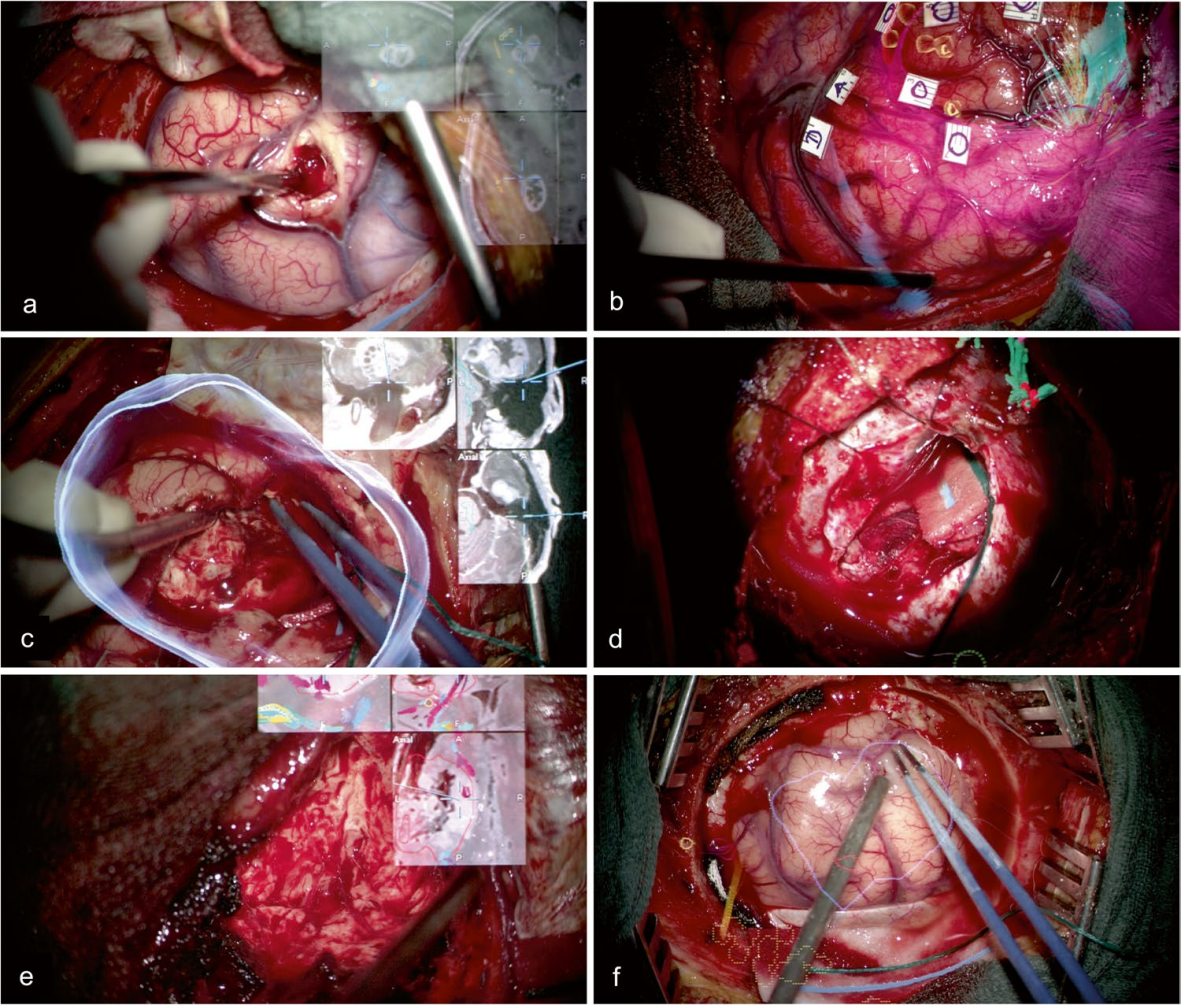
Case examples showing different views of in situ visualization: navigation PiP in a central tumor adjacent to the motor system (functional information, cortical hotspots as determined with nTMS, corticospinal tract) (**a**); volume overlay during awake language mapping in a left frontal tumor (tumor borders, nTMS-positive spots for language, most relevant subcortical tracts of the language system) (**b**); combination of volume overlay (tumor) and navigation PiP in an anaplastic astrocytoma right temporal-insular (**c**); target volume PiP in a pineal AVM (nidus, feeders, drainers) (**d**); image fusion of presurgical data and intraoperative MRI update for navigated resection of tumor remnant in an anaplastic oligodendroglioma (**e**); outline overlay in a right parietal anaplastic astrocytoma (tumor borders, nTMS-positive spots for motor, corticospinal tract) (**f**)

Only routine medical imaging data has been used in this study, including magnetic resonance imaging (MRI), diffusion tensor imaging (DTI), and brain mapping results from navigated transcranial magnetic stimulation (nTMS). The structural acquisitions on a 3 T MRI scanner (Siemens, Erlangen, Germany) included a T1 MPRAGE anatomical sequence 0.9 mm isotropic resolution, TR/TE 2300/2.32 ms, TI 900 ms, and flip angle 8 degree, for an acquisition time of 5:18 min. Diffusion data for tractography included 2 mm isotropic resolution whole brain acquisitions, TR/TE 7500/95 ms, 1 shell *b* value=1300 s/mm2 with 60 directions per shell. The scans were performed with a standard ep2d sequence, for an acquisition time of 5:47 min. The participating surgeons received a two-stage (basic and case-based) training in HUD control and microscope navigation. They performed segmentation of tumors and additional anatomical structures themselves, usually on the day of surgery; tractography and import of nTMS-positive spots, where applicable, were provided in advance by the same lab members who routinely transfer preoperative brain mapping results into the surgical planning software, and were reviewed by the surgeons in the final version of the plan. All surgical cases have been performed in a regular OR environment using a standard surgical microscope (OPMI Pentero/Kinevo 900, Carl Zeiss Meditec AG, Oberkochen) and the latest update of a commercial software for cranial planning and navigation (Cranial Navigation 3.1.4, Microscope Navigation 1.5.1, Image Fusion 4.0.0, Fibertracking 1.0.0, SmartBrush 2.6.0; Brainlab AG, Munich). Preset HUD views enabled by the microscope navigation software include augmented volumes/outlines of a target and probe’s eye (i.e., a reconstruction of the sectional imaging data within the focal plane) both as overlay and picture-in-picture (PiP), as well as navigation view in axial, coronal, and sagittal (ACS) orientation (PiP only). An illustrated case marks the combined view of target volumes and navigation PiP (see Fig. 1a–f). All cases have been documented by recordings of the microscope video stream.

### Study design and randomization

The main inclusion criterion for study cases was that there had to be expected added benefit from navigation information during at least one step of surgery (craniotomy, access route, identification of tumor borders, avoidance of risk structures). Between November 2017 and September 2018, a series of navigated brain tumor cases (*n*=92) with compatible imaging data, operated either with the AR-navigated microscope or conventional neuronavigation, has been analyzed prospectively. Cases have been assigned randomly to one of the groups unless the procedure times of included AR cases overlapped; with only one AR setup being available, subsequent cases if occurring had to be assigned to the control group (pseudorandomization). All cases with technical failures occurring at the beginning of surgery, with incomplete documentation and/or missing or corrupted microscope video, have been excluded from analysis (*n*=36, of which *n*=13 were initially assigned to the AR group and *n*=23 to the control group). Technical issues were the most important reason for exclusion of cases in the AR group, while incomplete data caused most case exclusions in the control group. The screen-based conventional neuronavigation system was available in all cases as a backup solution. Surgical resection time, duration/type/mode of AR, displayed objects (*n*, type), pointer-based navigation checks (*n*), and case-specific visual quality indicators (e.g., depth perception, accuracy) have been assessed based on participant observation in the OR and video analysis of the microscope recording (see Table 1). The usability and overall surgical usefulness of AR were rated by different neurosurgical experts (*n*=7) on a 5-point Likert scale (1=poor, 5=very good) during and after the intervention. Ethical approval has been obtained from the local IRB (EA1/037/16, updated version EA1/016/19), and written patient consent was collected prior to data acquisition. This study has been registered with the German Clinical Trials Register (DRKS00016955).

**Table 1:** Metrics for comparative intraoperative assessment of AR guidance and conventional neuronavigation in neurosurgery

Procedure	HUD view	Surgical task	Scientific evaluation
• Neuro-oncological • Neurovascular	• Target (volumes, outlines) • Navigation • Probe’s eye • Mixed (target+navigation) • Overlay/PiP	• Location and identification of pathology • Avoidance of risk structures • Surgical intervention (dissection, resection)	AR qualitative • Usability of HUD remote control • Effects of occlusion and distraction • Surgical depth perception • Stability of visualization • Accuracy of overlay • Color/complexity of visualization • Relevance for decision-making across surgical steps • Inter-individual visualization differences
			AR quantitative • Overall duration of AR/case • Duration of HUD views/case • Type of source data • Number/type of displayed objects • Number of fade-ins/fade-outs
			Non-AR quantitative • Frequency of pointer utilization • Type of source data • Number/type of displayed objects • Color/complexity of visualization
			AR/non-AR procedural • Overall duration of procedure and resection time • Number/type of adverse events • Type/location of pathology • Functional eloquence • Patient outcome (oncological, neurological)

### Quality assessment of visual information

**Fig. 2:**
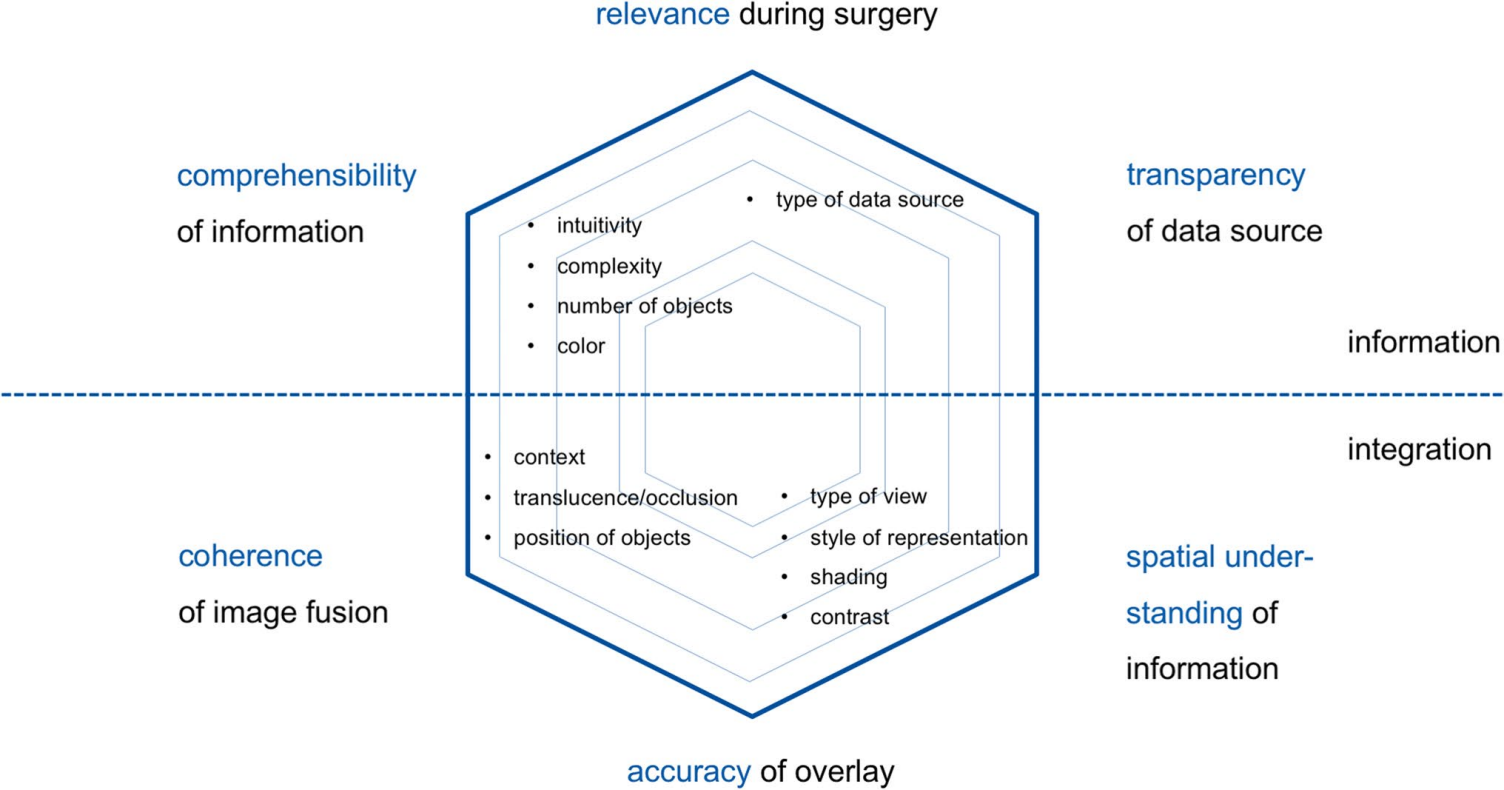
Selected quality dimensions of AR visualization in neurosurgery, grouped into general aspects of surgical information (upper part) and technical integration (lower part)

Following a literature research in the visualization quality of AR applications for neurosurgery and initial hands-on experience with the software, we defined the subsequent dimensions of visual representation as relevant for our investigation: depth perception and spatial understanding, style of 2D/3D representation (e.g., outlines, volume renderings), color coding, shading, translucence, and occlusion, contrast, type of (virtual) data, transparency of data source (quality, significance, limitations), type of view, number, position and complexity of displayed objects, overlay accuracy, coherence of image fusion and integration of contextual information, intuitivity and comprehensibility of AR scene, and relevance for surgical decision-making during intervention (see Fig. 2) [16, 21, 24, 25, 35]. Most of these items apply equally to data visualization in the conventional neuronavigation system and can thus be used for a comparative analysis.

### Statistical analysis

All data has been analyzed descriptively using the Statistical Package for Social Sciences (SPSS statistics version 27, 2020, IBM, Armonk/NY, USA). After correction for multiple testing using the Bonferroni test, two-tailed probability values of *P*<0.00625 were considered statistically significant.

## Results

Fifty-four patients undergoing 55 surgeries were included in the study (AR navigation *n*=39, conventional neuronavigation *n*=16). Mean patient age was 48.1 year (11–84; SD 15.8); 54.5% were male with a m/f ratio of 1.2:1. The lesions included 96.4% tumor and 3.6% vascular cases (AVM, CM). Dominant neuro-oncological lesions were 38.2% glioblastoma (GBM), 14.5% anaplastic astrocytoma (aAST), 9.1% metastasis (Met), 7.3% anaplastic oligodendroglioma (aODG), 7.3% oligodendroglioma (ODG), and 23.4% other, of which 29.1% were recurrences (see Table 2). 58.2% of the lesions were located in the left hemisphere, and 69.1% were considered partially or entirely deep-seated [33]. The percentage of deep-seated lesions in the AR group was 76.9% and 50% in the control group. The presurgically planned extent of resection (EOR) could be achieved in 89.1% of cases (89.7% of AR group, 87.5% of control group). A total of 79.5% of tumors in the AR group were considered locally infiltrative as opposed to 75% of pathologies in the control group. Supportive modalities for resection control (such as intraoperative MRI, intraoperative neuromonitoring, and fluorescence) have been used equally in both groups except for fluorescence lesion labeling (51.3% in AR group, 62.5% in control group).

**Table 2:** Case overview and characteristics of intraoperative visualization

No	Group	Sex/age	Diagnosis	Pathology	Lesion location	Data visualization
1	Interv	f44	ID	MNG	Frontobasal	MRI (tumor, vessels, nerves)
2	Contrl	f46	ID	GBM	Right parietal	MRI, DTI, nTMS (tumor, CST, M1)
3	Interv	f46	ID	GBM	Right parietal	MRI, DTI, nTMS (tumor, CST, M1)
4	Interv	f45	ID	dG	Left insular	MRI, DTI, nTMS (tumor, vessels, CST, M1, language network)
5	Interv	f34	ID	ODG	Ieft precentral	MRI, DTI, nTMS (tumor, CST, M1)
6	Interv	m36	ID	aODG	Left temporal	MRI (tumor)
7	Interv	f56	ID	aAST	Left central	MRI, DTI, nTMS (tumor, CST, M1)
8	Interv	f55	REC	aAST	Left insular	MRI, DTI (tumor, CST)
9	Interv	f40	ID	aODG	Left frontal	MRI, DTI, nTMS (tumor, language network)
10	Interv	f24	ID	GBM	Right frontal	MRI (tumor)
11	Contrl	f31	REC	GBM	Left parietal	MRI (tumor)
12	Interv	f48	ID	MNG	Right clinoid	MRI (tumor, vessels)
13	Contrl	f49	ID	GBM	Left insular	MRI (tumor)
14	Interv	f41	ID	CP	Sella	MRI (tumor)
15	Contrl	f67	ID	CM	Left precentral	MRI
16	Interv	m59	ID	dAST	Right frontal	MRI, DTI, nTMS (tumor, CST, M1)
17	Interv	m55	REC	GBM	Left temporal	MRI, DTI, nTMS (tumor, CST, M1, language network)
18	Interv	m44	ID	aAST	Right parietal	MRI, DTI, nTMS (tumor, CST, M1, language network)
19	Contrl	m78	ID	GBM	Right parietal	MRI, DTI (CST, language network)
20	Contrl	f38	REC	GBM	Right frontal	MRI, DTI (tumor, ventricle, language network)
21	Interv	m48	ID	AVM	Pineal	MRI (tumor, vessels)
22	Contrl	m43	REC	aODG	Left frontal	MRI, DTI (ventricle, language network)
23	Interv	m28	REC	aAST	Right temporal	MRI, DTI (tumor, CST)
24	Interv	m34	REC	aAST	Left frontal	MRI, DTI, nTMS (tumor, CST, M1)
25	Contrl	m55	ID	Met	Right occipital	MRI (tumor)
26	Interv	m59	ID	GBM	Right temporal	MRI (tumor)
27	Interv	f50	REC	GBM	Left parietal	MRI, DTI, nTMS (tumor, CST, M1)
28	Interv	m25	ID	Met	Right frontal	MRI
29	Contrl	m61	ID	Met	Right frontal	MRI
30	Interv	f58	REC	Met	Left central	MRI (tumor)
31	Interv	m37	ID	ODG	Left insular	MRI, DTI (tumor, language network)
32	Interv	f72	ID	GBM	Left parietal	MRI
33	Interv	m44	ID	aMNG	Left frontal	MRI (tumor)
34	Interv	m25	ID	aAST	Left frontal	MRI, DTI, nTMS (tumor, CST, M1, language network)
35	Interv	m29	ID	dAST	Left insular	MRI, DTI, nTMS (tumor, CST, M1)
36	Interv	m39	ID	GBM	Right temporal	MRI, DTI (tumor, CST, language network)
37	Interv	m36	REC	GBM	Right temporal	MRI (tumor)
38	Interv	m75	ID	Met	Left periventricular	MRI (tumor)
39	Contrl	m67	REC	GBM	Right temporal	MRI, DTI (tumor, CST)
40	Contrl	m57	ID	GBM	Left frontal	MRI (tumor)
41	Interv	f78	REC	atMNG	Left opercular	MRI (tumor)
42	Contrl	m11	ID	AVM	Left parietal	MRI
43	Interv	f41	ID	dG	Left insular	MRI, DTI (CST, language network)
44	Contrl	m32	REC	aODG	Left frontal	MRI, DTI (CST)
45	Contrl	m33	ID	aAST	Left parietal	MRI, DTI (tumor, CST)
46	Contrl	f51	ID	ODG	Multiple lesions	MRI, DTI (tumor, CST, language network)
47	Interv	f84	REC	atMNG	PCF	MRI (tumor)
48	Contrl	m52	ID	ODG	Right frontal	MRI, DTI (tumor, CST)
49	Interv	m65	REC	GBM	Left parietal	MRI, DTI (CST)
50	Interv	m62	ID	GBM	Left central	MRI, DTI, nTMS (CST, M1)
51	Interv	m51	ID	GBM	Left temporal	MRI (tumor)
52	Interv	f66	ID	GBM	Left temporal	MRI, DTI, nTMS (tumor, language network)
53	Interv	f29	REC	GBM	Left postcentral	MRI
54	Interv	m40	ID	aAST	Right temporal	MRI, DTI (tumor, CST)
55	Interv	f70	ID	GBM	Left temporal	MRI, DTI, nTMS (tumor, CST, M1, language network)

Abbreviations: *aAST*, anaplastic astrocytoma; *aMNG*, anaplastic meningioma; *aODG*, anaplastic oligodendroglioma; *atMNG*, atypical meningioma; *AVM*, arterio-venous malformation; *CM*, cavernoma; *CP*, craniopharyngioma; *CST*, corticospinal tract; *dAST*, diffuse astrocytoma; *dG*, diffuse glioma; *GBM*, glioblastoma; *ID*, initial diagnosis; *M1*, primary motor cortex; *Met*, metastasis; *MNG*, meningioma; *PCF*, posterior cranial fossa; *REC*, recurrence

A total of 14.5% of patients (*n*=8; 17.9% of AR group, 6.3% of control group) had a transient deficit after surgery (visual, sensible, motor, speech, mnestic), 12.7% of patients (*n*=7; 12.8% of AR group, 8.1% of control group) had a permanent new deficit after 3 months (visual, motor, speech), and 9.1% of patients (*n*=5; 7.7% of AR group, 12.5% of control group) improved postoperatively (motor, visual, aphasia, mnestic).

Planning, preparation, and calibration of the microscope navigation connection for AR visualization were added on average 11.1 min (4–38; SD 6.4) surgical time per case. We could not identify any surgical complications related to HUD-based AR utilization.

### Evaluation of AR visualization

**Fig. 3:**
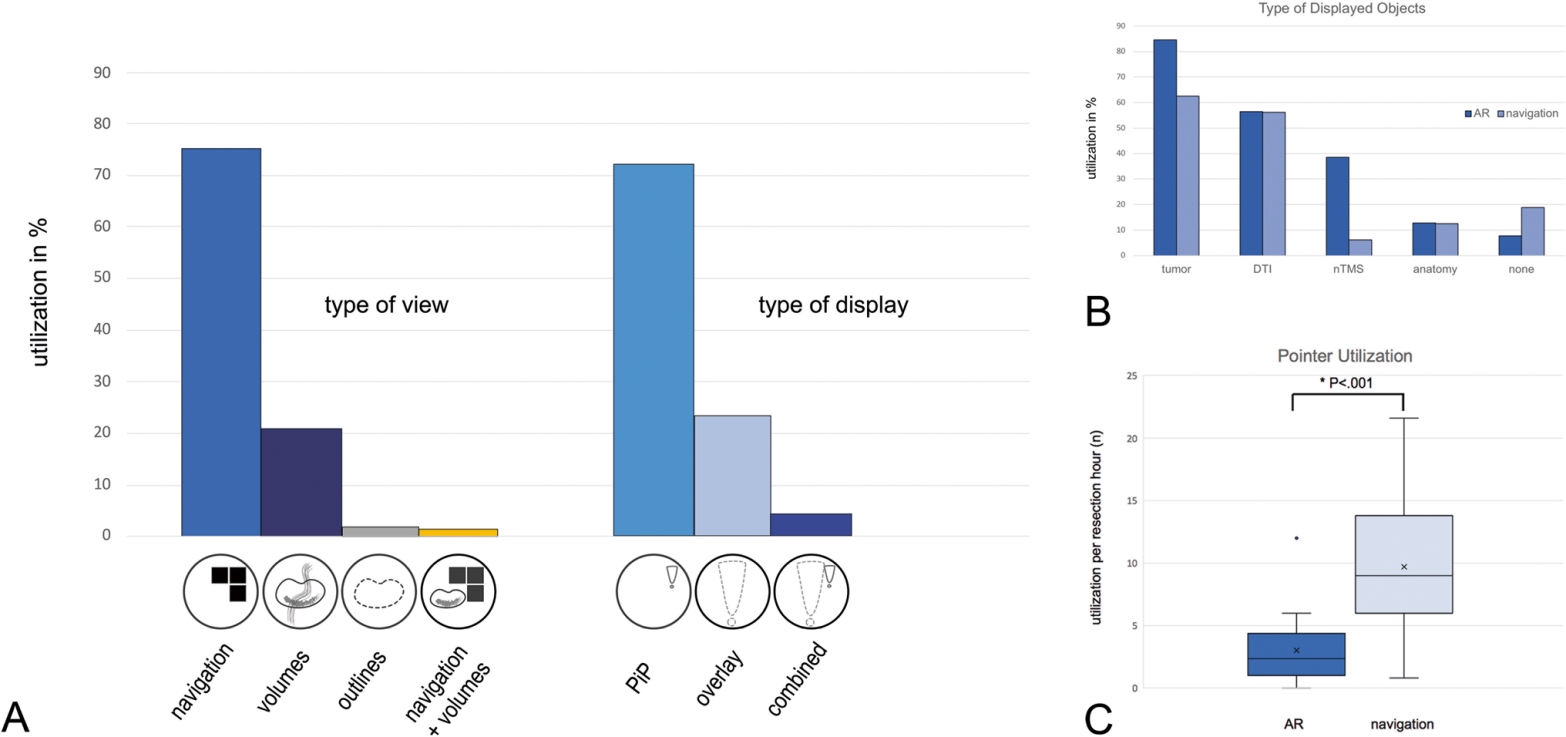
Utilization of AR visualization during surgery: types of HUD view and display (**a**), frequency of displayed objects (**b**), and comparison of pointer-based navigation per hour of resection between AR and neuronavigation group (**c**)

In the AR group, AR has been utilized in 44.4% (mean 32.2 min) of total resection time (mean 72.5 min). In 43.6% of cases, important anatomical structures have been partially or completely obscured by AR information at least once during surgery. Frequently used HUD display modes were navigation view (75.7%) and target volume mode (20.1%), displayed predominantly as PiP (72.5%) and occasionally as overlay (23.3%), rarely as a combination of both (4.2%) (see Fig. 3a). In navigation view, standard ACS injection (axial, coronal, and sagittal sectional imaging) was used primarily as continuous orientation display. Compared to conventional neuronavigation systems, a combined display of different MRI sequences (e.g., T1 with contrast enhancement and T2 FLAIR) was not feasible with the current software generation.

On average, two navigation objects (0–4; SD 1.1) have been displayed in the AR group compared to 1.4 objects in the conventional neuronavigation group (0–3; SD 1) (*U*=218.000, *P*=0.070; CI 95). Additional tumor segmentation was the most frequent object type in both groups (84.6% in AR vs. 62.5% in navigation), followed by tractography for motor and language (56.4% in AR vs. 56.2% in navigation), nTMS data for cortical hotspots (38.5% in AR vs. 6.2% in navigation), and anatomical structures such as vessels, cranial nerves, or ventricles (12.8% in AR vs. 12.5% in navigation). MRI-only navigation view has been utilized in 7.7% (AR) and 18.8% of cases (conventional neuronavigation), respectively (see Fig. 3b). In non-AR cases, pointer-based navigation checks were associated with frequent workflow interruptions (5–28 s each). The effort of pointer utilization was significantly reduced with 2.6×/resection hour (0–12; SD 2.53) in the AR group versus 9.7×/resection hour (0.8–21.6; SD 5.6) in the control group (*U*=557.500, *P*<0.001; CI 95) (see Fig. 3c). In AR cases, the navigation pointer has been used mainly for position verification (early stages of surgery) and for estimation of brain shift (advanced stages of surgery).

The dimensions of visualization quality have been rated equally to poorly in the navigated microscope (*n*=34) compared to conventional neuronavigation (*n*=12). Notable differences were found in spatial understanding of information (median 3.0 in AR vs. 5.0 in navigation, median test *P*<0.001; CI 95), visual accuracy of overlay (median 3.0 in AR vs. 5.0 in navigation, median test *P*<0.001; CI 95), visual transparency of data source (median 3.0 in both groups, median test *P*=0.002, CI 95, chi-square test 9.337), and visual comprehensibility (median 3.0 in AR vs. 4.0 in navigation, median test *P*<0.001, chi-square test 25.314). In comparison, coherence of image fusion (median 3.0 in both groups, median test *P*=0.060, CI 95, chi-square test 3.527) and relevance of visualization (median 4.0 in AR vs. 5.0 in navigation, median test *P*=0.559, CI 95, chi-square test 0.342) showed no significant differences.

When verified with the navigation pointer and a sterile paper ruler by the surgeon (*n*=9), offset range in AR visualization was 0–7 mm. A total of 66.7% of participants stated that AR visualization was helpful for their surgical case; 76.9% of those cases were deep-seated lesions. The surgeons who found AR visualization helpful displayed two navigation objects on average and showed in 84.6% a fine command of the HUD visualization. A total of 65.4% of them indicated that their focus view remained unblocked during surgery, using navigation view as PiP display in 88.6% of the cases, while 30.8% encountered technical issues during preparation and/or surgery. The mean pointer utilization was reduced in this subgroup (2.2×/resection hour), whereas the mean AR utilization was increased (39.5 min).

In the qualitative assessment of individual decision-making and surgical preferences which has been conducted in interviews during and after surgery, we collected a broad range of aspects for both preference and abandonment of the technique. Recurring issues included 2D vs. 3D visualization of surgically relevant information (e.g., for better understanding of topographical anatomy and tumor borders) and the visual integration in the field of view (e.g., blending vs. occlusion) (for a structured summary see Table 3 of the supplementary material).

## Discussion

In this study, we performed a clinical evaluation of HUD-based AR visualization in cranial surgery, using a commercially available software for microscope navigation. We found that the negative impact of this technique on clinical workflow is low as it uses components already established in surgical routine. Moreover, we were able to quantify the practical facilitation of neuronavigation when using AR navigation view instead of pointer-based techniques. Particularly in the inexperienced user, AR visualization can be disruptive as neurosurgeons need to learn how to read and apply the information. In spite of its clinical feasibility, AR utilization in neurosurgical routine requires further investigation regarding case selection, visualization, and technical limitations, as will be discussed in the following sections.

### Case selection and clinical application

The identification of eligible cases differs in different AR technologies; previous studies reported, for instance, superficial brain tumors being particularly suitable for projection techniques [20] and small deep-seated lesions being ideal for HUD-based AR [22], despite a reduced rate of accuracy in the depth [33].

Our findings support that AR guidance is more helpful in deep-seated lesions (>1 cm from the cortex), namely, during the targeting of small structures, the consulting of tumor borders, or the identification of functionally eloquent areas in conjunction with intraoperative neuromonitoring. While both neuro-oncological and neurovascular cases are frequently reported in the literature as predominant application areas, the majority of our cases turned out to be intra-axial tumors. However strongly articulated as a clinical demand, the support of neurovascular cases using the existing software application was restricted by prevailing technical limitations for the duration of this study. Here, future solutions need to incorporate the detailed vascular architecture surrounding the surgical target (e.g., in AVMs), preferably containing information on blood flow (i.e., direction, velocity) and oxygen concentration (i.e., arterial, venous).

In several studies, AR is frequently used for preoperative craniotomy as well as skin incision planning and stated as helpful or beneficial [2–5, 20, 33] despite the fact that a precise advantage or a superiority of AR compared to conventional point-reference navigation is missing. In particular, the role of perspective, distortion, and potentially imperfect motion parallax [34] is underestimated in terms of impact on registration and navigation accuracy. In this study, participants used AR visualization throughout the intervention, requesting especially during initial tumor resection more detailed topographical information. Surgeons who were most comfortable with the navigation view used AR visualization to guide the entire tumor removal, in some cases with an updated image, provided by intraoperative imaging (MRI). Overlay visualization, however, was stated to be useful for intermittent overview and identification of structures within the focal plane, hence showing more of an educational potential for surgical assistants and team.

In general, AR visualization in the microscope HUD can help account for registration inaccuracy and brainshift [1, 22] using the re-registration feature based on — usually superficial — intracranial structures. In our study, surgeons who used this feature infrequently after durotomy found it helpful.

### Comparison with conventional neuronavigation

In the minimal version of HUD-based visualization, the navigated microscope constitutes an integrated pointer without exploiting the potential of an AR display. As has been discussed before, AR visualization can contribute to focused information flow during surgery, reducing alternate viewing directions and attention shifts [7, 30] as well as changing of instruments. However, obvious workflow facilitations such as pointer-free navigation and fade-in display of surgical information were accompanied in our study by partial blocking of the surgical field and impaired depth assessment. This also extends to brain surface structures used for anatomical orientation. Accordingly, most participants preferred a peripheral display of information over AR visualization in the focus level due to visual occlusion and reported distraction effects [12]. In addition, the direct application of visual information onto the surgical site — without changing the source data quality — promoted a more critical approach towards the process of data guidance itself as well as decision-making in data visualization. Visualization habits (type, number, and color of objects) established in conventional neuronavigation were more or less reproduced in AR navigation with two exceptions: some surgeons tended to do some extra planning for AR guidance in the OR, and they changed the tumor color from red to blue or green as to render it more distinguishable from the surgical site. We expect that with enhanced visualization technology, dedicated concepts for multimodal neuronavigation will evolve in general.

### Dimensions of visual quality

**Fig. 4:**
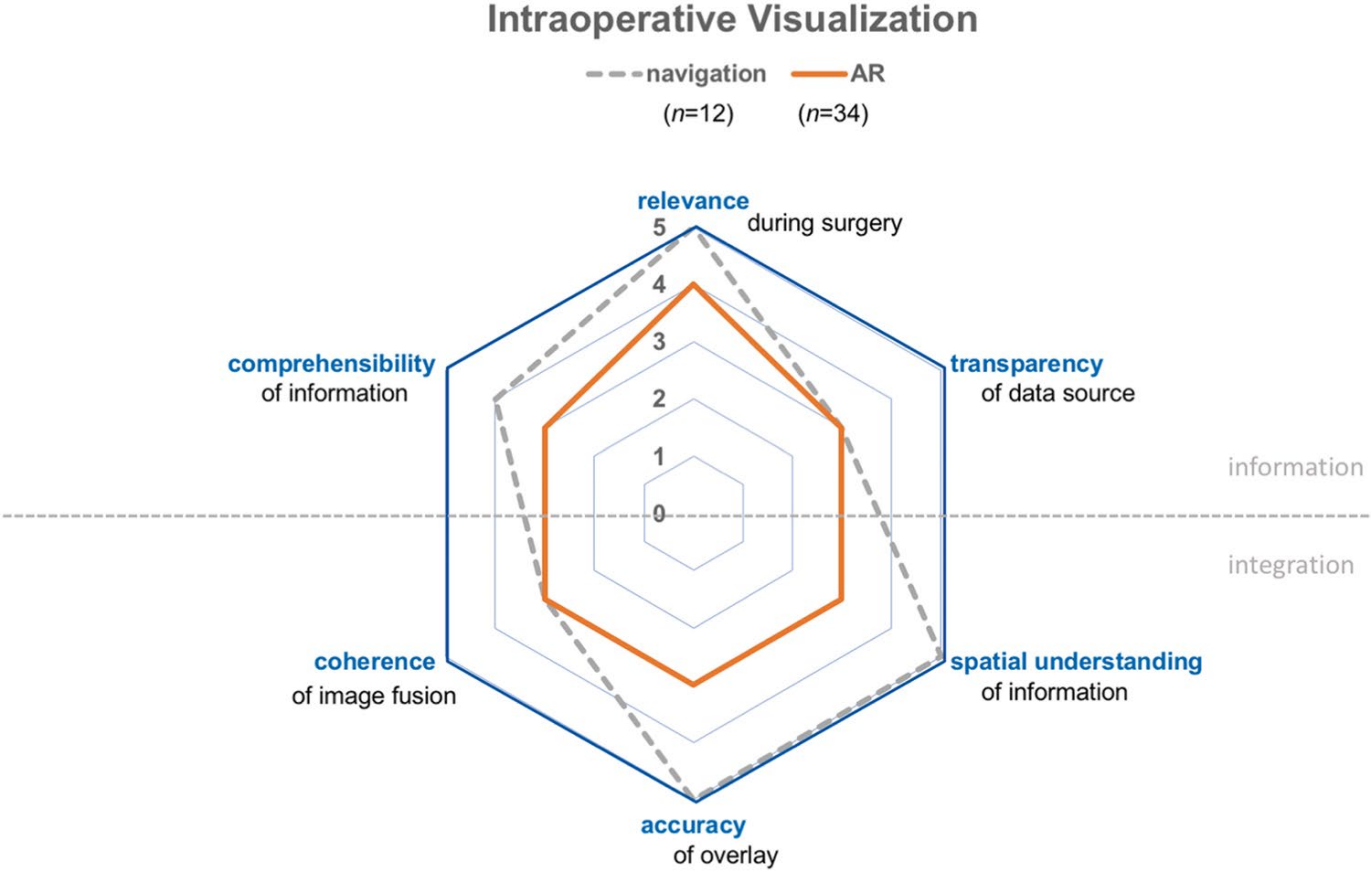
Overall quality of intraoperative visualization based on surgeon ratings for neuronavigation (dotted line) and AR (solid line). All graphs and illustrations have been created using Adobe Illustrator CC 2017.0.2

To date, there are no established standards for measuring the quality of AR visualization in neurosurgery. Two publications suggest a strategical framework for device assessment focusing on technical setup, display types, and the processing of imaging data [25, 35]. Based on our case experience from this study, we are, however, able to expand on our initial model of visualization quality (Figs. 2 and 4 and Table 3 of the supplementary material).

The selection of appropriate information depends on several contributing factors, such as availability (i.e., imaging modality, slice thickness, volumetric data, special sequences) and individual command of software functionality and personal habits. We observed no difference in the type of data used for intraoperative visualization between AR guidance and conventional neuronavigation. Accordingly, AR visualization quality largely depends on the quality of the underlying data [21], usually CT or MRI, and successful image fusion. Image resolution and object segmentation, in particular, pose a challenge for microscope injection when working at high magnification, which is usually the case in neurosurgery: small caliber vessels and nerves of interest are often poorly delineated in the source data, while larger structures appear rough and bulky when superimposed to the surgical site. In case of interpretation difficulties or barely demarcating anatomical structures in the sectional imaging data, there is no added benefit with AR visualization.

Despite being the favored view in our study, the AR navigation view does not reveal context information outside the currently displayed sectional planes; it requires further interaction, such as change of focal plane, to provide visualized information above and below the actual resection plane, which can be crucial in minimally invasive or keyhole approaches [43].

While the current software generation introduced more naturalistic 3D volume injection in navigated microscopes, any conveyed object depth information (e.g., of adjacent white matter fibertracking in tumor targets) has been yet rated unintuitive by the surgeons. As the visualization remains always on top of the AR scene, the placement of objects does not follow context or background features, and volumetric data does not properly merge with the scene [21]. Consequently, reliable information on tool-target distance is still lacking. Standard depth cues, such as perspective (stereo disparity), object size, solidity, and detail, were further susceptible to one particular requirement of the HUD-based AR visualization investigated in this study, which is the perpendicular axis of microscope lens and focal plane. As a result of diverging angles to plane, surgeons complained about visual artifacts in the AR scene (distortion, perceived offset of tissue boundaries). However, target volumes (as overlay) are a promising feature, given that registration and calibration errors are low. They can indicate the maximum extension of surgically relevant structures in relation to the focal plane and include three-dimensional information on adjacent topography, which is otherwise only provided in volumetric visualization of presurgical planning data. Analogue to our experience with conventional neuronavigation, high numbers of displayed objects (*n*>3) cannot be recommended as they cause distraction and informational overload (visual fatigue). Regrettably, current commercial AR visualization of volumes still remains restricted by at least a few unsolved issues in transparency, shading, and visual occlusion, preventing a more effective merging of physical and virtual information. Eliminating the necessity of a multiple screen scenario during consultation of presurgical planning data, they can best be used as a recapitulatory step before switching to more instructive views (e.g., navigation view). With the ongoing technical improvements in digital visualization, we expect a further and profound change towards better integrated AR information that can be used comfortably throughout surgery at different scales and degree of detail.

### Technical limitations

As we encountered a comparably high number of technical issues (e.g., lost connection, missing or incomplete data) in the course of this study, we suggest implementing better user guidance in the software interface for AR navigation. Additionally, built-in features for measuring registration error and accuracy of overlay [19] would be desirable. While readily available in most hospitals, microscope-based AR systems are nevertheless potentially impractical because of the optics of the surgical microscope itself [35]. Stereoscopic AR visualization is limited as the microscope captures a monoscopic bidimensional view of the surgical field, thus preventing the three-dimensional virtual image from merging with the real scene. Here, we expect novel impulses and solutions from emerging digital 3D exoscope technology. Besides, a combination of fluorescence-guided surgery for the detection of tumor remnants and AR information overlay with the purpose of further guidance and mutual validation is technically impossible at this stage.

### Limitations of this study

A major limitation of this study is the imbalance of the two groups after randomization, which can be largely explained by not using a 1:1 allocation at the beginning followed by different reasons for subsequent case exclusion (technical issues in the AR group, incomplete data in the navigation group).

Navigating the focal point of the surgical microscope does not exploit the full potential of AR visualization in surgery. Microscope navigation is one case study among many which can contribute to the understanding of intraoperative visualization requirements.

Most cases of our series were intracranial tumors. We included only two neurovascular pathologies (AVM, CM) for the majority of those cases at our institution is usually treated relying on anatomical landmarks (without neuronavigation) and/or intraoperative imaging. Particularly in AVMs, we expect considerable improvements with an upcoming software update allowing for better discrimination of arterial feeders, venous drainage, and nidus structures based on high-resolution three-dimensional, partially automated vessel segmentation. A randomized study with high case numbers will be needed for validation of presented findings; however, this might prove difficult as it involves abandoning the established gold standard of neuronavigation in potentially complex procedures with users less experienced in AR navigation. Accordingly, there are no prospective studies showing a significant difference between AR-aided surgeries versus navigation-guided procedures regarding morbidity, mortality, and clinical effectiveness (EOR vs. functional outcome). Given the differences in group size and distribution of pathologies, the clinical results of this study can only be suggestive in regard of certain tendencies. A considerable limitation of current AR navigation using visual overlay is the visualization offset, which can be even more distracting at high magnification than comparable offset in pointer-based neuronavigation. Here, a standardized investigation across technical setups, different pathologies, and surgical approaches will be necessary. As for the investigation of intraoperative visualization standards, a multicenter comparison could provide further insights in terms of applicability and scalability of requirements and recommendations. Future work should include the visual standardization across applications in particular in order to contribute substantial clinical data to the ongoing assessment of augmented, mixed, and virtual reality techniques in neurosurgery (and potentially beyond).

The evaluation of AR in exoscopic surgery is subject of a follow-up study at our institution.

## Conclusions

HUD-based AR visualization in routine brain tumor surgery is clinically feasible and safe. Its most salient feature is the pointer-free navigation during tissue preparation and tumor removal, minimizing the current ergonomic hindrances, which require surgeons to look alternately at multiple displays. While the technical workflow is at large compliant with daily surgical routine, the visualization quality still impacts surgical cognitive load and performance. The new software generation offers a more “realistic” yet clearly distinguishable style of visualization for surgically relevant information. Known restrictions of the technology are due to, firstly, the overlay of working and viewing area; secondly, the lack of stereoscopic three-dimensional depth information in the AR scene; and thirdly, potential visualization offset caused by MRI data resolution, registration errors, and brainshift during surgery. Factors promoting the application of AR navigation, as identified in this study, are deep-seated lesions and peripheral navigation view including two displayed essential objects (usually tumor and adjacent risk structures) in a trained user. Personal surgical preferences affect the utilization of the technique; since the navigated microscope is an extension of conventional neuronavigation, the habits of use and visualization style are largely comparable.

## Supplementary Information

Below is the link to the electronic supplementary material.

Supplementary file1 (DOCX 16 KB)

Supplementary file2 (DOC 37 KB)

Supplementary file3 (DOC 221 KB)

## References

[1] Asano K, Katayama K, Kakuta K, Oyama K, Ohkuma H (2017). Assessment of the accuracy and errors of head-up display by an optical neuronavigation system in brain tumor surgery. Oper Neurosurg (Hagerstown).

[2] Cabrilo I, Bijlenga P, Schaller K (2014) Augmented reality in the surgery of cerebral aneurysms: a technical report. Neurosurgery 10 Suppl 2:252–260; discussion 260–261 10.1227/NEU.0000000000000328 24594927

[3] Cabrilo I, Bijlenga P, Schaller K (2014). Augmented reality in the surgery of cerebral arteriovenous malformations: technique assessment and considerations. Acta Neurochir (Wien).

[4] Cabrilo I, Schaller K, Bijlenga P (2015). Augmented reality-assisted bypass surgery: embracing minimal invasiveness. World Neurosurg.

[5] Cabrilo I, Sarrafzadeh A, Bijlenga P, Landis BN, Schaller K (2014). Augmented reality-assisted skull base surgery. Neurochirurgie.

[6] Carl B, Bopp M, Benescu A, Saß B, Nimsky C (2020). Indocyanine green angiography visualized by augmented reality in aneurysm surgery. World Neurosurg.

[7] Carl B, Bopp M, Saß B, Pojskic M, Voellger B, Nimsky C (2020). Spine surgery supported by augmented reality. Global Spine J.

[8] Cho J, Rahimpour S, Cutler A, Goodwin CR, Lad SP, Codd P (2020). Enhancing reality: a systematic review of augmented reality in neuronavigation and education. World Neurosurg.

[9] Contreras López WO, Navarro PA, Crispin S (2018). Intraoperative clinical application of augmented reality in neurosurgery: a systematic review. Clin Neurol Neurosurg.

[10] Besharati Tabrizi L, Mahvash M (2015). Augmented reality-guided neurosurgery: accuracy and intraoperative application of an image projection technique. J Neurosurg.

[11] Deng W, Li F, Wang M, Song Z (2014). Easy-to-use augmented reality neuronavigation using a wireless tablet PC. Stereotact Funct Neurosurg.

[12] Dixon BJ, Daly MJ, Chan HHL, Vescan A, Witterick IJ, Irish JC (2014). Inattentional blindness increased with augmented reality surgical navigation. Am J Rhinol Allergy.

[13] Drouin S, Kersten-Oertel M, Louis Collins D, Linte CA, Yaniv Z, Fallavollita P (2015). Interaction-based registration correction for improved augmented reality overlay in neurosurgery. Augmented environments for computer-assisted interventions.

[14] Edwards PJ, Hawkes DJ, Hill DL, Jewell D, Spink R, Strong A, Gleeson M (1995). Augmentation of reality using an operating microscope for otolaryngology and neurosurgical guidance. J Image Guid Surg.

[15] Edwards PJ, King AP, Hawkes DJ (1999). Stereo augmented reality in the surgical microscope. Stud Health Technol Inform.

[16] Guha D, Alotaibi NM, Nguyen N, Gupta S, McFaul C, Yang VXD (2017). Augmented reality in neurosurgery: a review of current concepts and emerging applications. Can J Neurol Sci.

[17] Gumprecht HK, Widenka DC, Lumenta CB (1999) BrainLab VectorVision Neuronavigation system: technology and clinical experiences in 131 cases. Neurosurgery 44(1):97–104; discussion 104–105 10.1097/00006123-199901000-00056 9894969

[18] Grimson WL, Ettinger GJ, White SJ, Lozano-Perez T, Wells WM, Kikinis R (1996). An automatic registration method for frameless stereotaxy, image guided surgery, and enhanced reality visualization. IEEE Trans Med Imaging.

[19] Holloway RL (1997). Registration error analysis for augmented reality. Presence Teleoperators and Virtual Environments.

[20] Inoue D, Cho B, Mori M (2013). Preliminary study on the clinical application of augmented reality neuronavigation. J Neurol Surg A Cent Eur Neurosurg.

[21] Kalkofen D, Sandor C, White S, Schmalstieg D, Furht B (2011). Visualization techniques for augmented reality. Handbook of Augmented Reality.

[22] Kantelhardt SR, Gutenberg A, Neulen A, Keric N, Renovanz M, Giese A (2015). Video-assisted navigation for adjustment of image-guidance accuracy to slight brain shift. Oper Neurosurg (Hagerstown).

[23] Kato A, Yoshimine T, Hayakawa T, Tomita Y, Ikeda T, Mitomo M, Harada K, Mogami H (1991). A frameless, armless navigational system for computer-assisted neurosurgery. Technical note J Neurosurg.

[24] Kersten-Oertel M, Gerard I, Drouin S, Mok K, Sirhan D, Sinclair DS, Collins DL (2015). Augmented reality in neurovascular surgery: feasibility and first uses in the operating room. Int J Comput Assist Radiol Surg.

[25] Kersten-Oertel M, Jannin P, Collins DL (2013). The state of the art of visualization in mixed reality image guided surgery. Comput Med Imaging Graph.

[26] King AP, Edwards PJ, Maurer CR (2000). Stereo augmented reality in the surgical microscope. Presence: Teleoperators and Virtual Environments.

[27] Kockro RA, Tsai YT, Ng I, Hwang P, Zhu C, Agusanto K, Hong LX, Serra L (2009). Dex-ray: augmented reality neurosurgical navigation with a handheld video probe. Neurosurgery.

[28] Kosterhon M, Gutenberg A, Kantelhardt SR, Archavlis E, Giese A (2017). Navigation and image injection for control of bone removal and osteotomy planes in spine surgery. Oper Neurosurg (Hagerstown).

[29] Lee C, Wong GKC (2019). Virtual reality and augmented reality in the management of intracranial tumors: a review. J Clin Neurosci.

[30] Léger É, Drouin S, Collins DL, Popa T, Kersten-Oertel M (2017). Quantifying attention shifts in augmented reality image-guided neurosurgery. Healthc Technol Lett.

[31] Liu T, Tai Y, Zhao C, Wei L, Zhang J, Pan J, Shi J (2020) Augmented reality in neurosurgical navigation: a survey. Int J Med Robot e2160 10.1002/rcs.2160 32890440

[32] Maciunas RJ, Berger MS, Copeland B, Mayberg MR, Selker R, Allen GS (1996). A technique for interactive image-guided neurosurgical intervention in primary brain tumors. Neurosurg Clin N Am.

[33] Mascitelli JR, Schlachter L, Chartrain AG, Oemke H, Gilligan J, Costa AB, Shrivastava RK, Bederson JB (2018). Navigation-linked heads-up display in intracranial surgery: early experience. Oper Neurosurg (Hagerstown).

[34] Maurer CR, Sauer F, Hu B (2001). Augmented-reality visualization of brain structures with stereo and kinetic depth cues: system description and initial evaluation with head phantom. Proceedings of SPIE Medical Imaging.

[35] Meola A, Cutolo F, Carbone M, Cagnazzo F, Ferrari M, Ferrari V (2017). Augmented reality in neurosurgery: a systematic review. Neurosurg Rev.

[36] Mikhail M, Mithani K, Ibrahim GM (2019). Presurgical and intraoperative augmented reality in neuro-oncologic surgery: clinical experiences and limitations. World Neurosurg.

[37] Milgram P, Kishino F (1994). A taxonomy of mixed reality visual displays. Ieice T Inf Syst.

[38] Perwög M, Bardosi Z, Diakov G, Jeleff O, Kral F, Freysinger W (2018). Probe versus microscope: a comparison of different methods for image-to-patient registration. Int J CARS.

[39] Roberts DW, Strohbehn JW, Hatch JF, Murray W, Kettenberger H (1986). A frameless stereotaxic integration of computerized tomographic imaging and the operating microscope. J Neurosurg.

[40] Roessler K, Ungersboeck K, Aichholzer M, Dietrich W, Goerzer H, Matula C, Czech T, Koos WT (1998). Frameless stereotactic lesion contour-guided surgery using a computer-navigated microscope. Surg Neurol.

[41] Rychen J, Goldberg J, Raabe A, Bervini D (2020). Augmented reality in superficial temporal artery to middle cerebral artery bypass surgery: technical note. Oper Neurosurg (Hagerstown).

[42] Sielhorst T, Feuerstein M, Navab N (2008). Advanced medical displays: a literature review of augmented reality. Journal of Display Technology.

[43] Spetzger U, Laborde G, Gilsbach JM (1995). Frameless neuronavigation in modern neurosurgery. Minim Invasive Neurosurg.

[44] Toyooka T, Otani N, Wada K, Tomiyama A, Takeuchi S, Fujii K, Kumagai K, Fujii T, Mori K (2018). Head-up display may facilitate safe keyhole surgery for cerebral aneurysm clipping. J Neurosurg.

[45] Watanabe E, Watanabe T, Manaka S, Mayanagi Y, Takakura K (1987). Three-dimensional digitizer (neuronavigator): new equipment for computed tomography-guided stereotaxic surgery. Surg Neurol.

