# Supplementary material for: Augmented reality visualization in brain lesions: a prospective randomized controlled evaluation of its potential and current limitations in navigated microneurosurgery
Source: Acta Neurochir (Wien). 2021 Dec 13;164(1):3–14. doi: 10.1007/s00701-021-05045-1 (PMC8761141; doi:10.1007/s00701-021-05045-1)
Supplement: Supplementary file 1 — Supplementary file1 (DOCX 16 KB) [file 701_2021_5045_MOESM1_ESM.docx]

Table 3: Advantages and disadvantages of the different AR visualization types and modes [supplementary material]

| **visualization parameter** | **advantages** | **disadvantages** |
| --- | --- | --- |
| target volumes (type) | - more naturalistic 3D volumes - providing topographical presurgical planning information - direct matching with surface structures | - depending on resolution and degree of detail in source data - depth information often uncertain - limited number of objects - short-term utilization during surgery |
| outlines (type) | - indicating maximum extension of sub-surface structures - less occlusion of field of view - long-term utilization during surgery | - depth information often uncertain - type of object often unclear - crossing and overlay of lines in multiple objects |
| navigation view (type) | - ›always on‹ real-time navigation information within field of view - less occlusion of field of view - combination with object segmentation - view as in conventional neuronavigation - long-term utilization during surgery | - no context information beyond currently displayed sectional planes - risk structures often beyond focal plane (interaction required) - depending on quality of MRI sequence - no combination of different sequences |
| probe’s eye (type) | - direct matching of sectional navigation view and surgical site in correct scale | - depending on resolution and degree of detail in source data - no context information beyond current sectional plane - occlusion of field of view - short-term utilization during surgery |
| target volumes + navigation view (type) | - both focal and global orientation - direct matching with surface structures | - occlusion of field of view - short-term utilization during surgery |
| overlay display (mode) | - direct matching with anatomical/ pathological structures - more naturalistic 3D volumes - indicating maximum extension of sub-surface structures - enabling estimation of brainshift | - no fusion with context features - no reliable tool-target distance - potential artifacts caused by visual distortion/varying angles to resection plane - depth information often uncertain - limited number of objects - occlusion of field of view - short-term utilization during surgery |
| picture-in-picture (mode) | - all-in-one display of surgically relevant information - less occlusion of field of view - long-term utilization during surgery | - no realistic scale - no context information - no relation to physical depth of resection cavity |
| picture-in-picture + overlay display (mode) | - both focal and global orientation | - occlusion of field of view - short-term utilization during surgery |
