# Supplementary material for: Augmented reality visualization in brain lesions: a prospective randomized controlled evaluation of its potential and current limitations in navigated microneurosurgery
Source: Acta Neurochir (Wien). 2021 Dec 13;164(1):3–14. doi: 10.1007/s00701-021-05045-1 (PMC8761141; doi:10.1007/s00701-021-05045-1)
Supplement: Supplementary file 2 — Supplementary file2 (DOC 37 KB) [file 701_2021_5045_MOESM2_ESM.doc]

**CONSORT 2017 NPT Flow Diagram**

**Allocation**

**Enrollment**

Assessed for eligibility (*n*=92)

Boutron I, Altman DG, Moher D, Schulz KF, Ravaud P, for the CONSORT NPT Group. CONSORT Statement for Randomized Trials of Nonpharmacologic Treatments: A 2017 Update and a CONSORT Extension for Nonpharmacologic Trial Abstracts. Annals of Internal Medicine. 2017;167(1):40. doi:10.7326/M17-0046

**Analysis**

**Follow-Up**

Allocated to intervention (AR navigation) (*n*=52)

♦ Received allocated intervention: 39

♦ Excluded after allocation due to missing data or technical issues: 13

Analysed (*n*=39)
♦ Excluded from analysis (*n*=0)

Excluded (*n*=1)

♦  Not meeting inclusion criteria: 1

♦  Declined to participate: 0

Analysed (*n*=16)
♦ Excluded from analysis (*n*=0)

Lost to follow-up (*n*=0)

Allocated to standard (conventional neuronavigation) (*n*=39)

♦ Received allocated intervention: 16

♦ Excluded after allocation due to missing data or technical issues: 23

Randomly assigned (*n*=91)

Surgeons

Surgeon 1: 2 patients

Surgeon 2: 1 patient

Surgeon 3: 4 patients

Surgeon 4: 1 patient

Surgeon 5: 3 patients

Surgeon 6: 3 patients

Surgeon 7: 2 patients

Surgeons

Surgeon 1: 12 patients

Surgeon 2: 6 patients

Surgeon 3: 10 patients

Surgeon 4: 7 patients

Surgeon 5: 2 patients

Surgeon 6: 1 patient

Surgeon 7: 1 patient

Lost to follow-up (*n*=0)
